# Impact of Vascular Access Flow Suppression Surgery on Cervical Artery Circulation: A Retrospective Observational Study

**DOI:** 10.3390/jcm13030641

**Published:** 2024-01-23

**Authors:** Koji Hashimoto, Makoto Harada, Yosuke Yamada, Taro Kanno, Yutaka Kanno, Yuji Kamijo

**Affiliations:** 1 Department of Nephrology, Shinshu University School of Medicine, 3-1-1 Asahi, Matsumoto 390-8621, Japan; khashi@shinshu-u.ac.jp (K.H.); tokomadaraha724@gmail.com (M.H.); yamada19860603@yahoo.co.jp (Y.Y.); 2 Kanno Dialysis and Vascular Access Clinic, 2-17-5 Tsukama, Matsumoto 390-0821, Japan; tarosuke0519@yahoo.co.jp (T.K.); kannohd.k-dac@go.tvm.ne.jp (Y.K.)

**Keywords:** hemodialysis, vascular access, high-flow access, cardiovascular events, subclavian steal syndrome

## Abstract

Vascular access (VA) flow suppression surgery augments VA flow resistance and can increase other circulation flows hindered by high-flow VA. However, whether VA flow suppression surgery affects cervical circulation has rarely been reported. We aimed to determine the effect of VA flow suppression surgery on the cervical circulation in patients with high-flow VA. This single-center, retrospective, observational study included 85 hemodialysis patients who underwent VA flow suppression surgery at the Kanno Dialysis and Access Clinic between 2009 and 2018. Blood flow in the VA, bilateral vertebral arteries, and common carotid artery was measured before and after VA flow suppression surgery. The VA flow decreased from 1548 mL/min to 693 mL/min postoperatively. The flow of the vertebral artery on the VA side increased from 55 mL/min to 81 mL/min. The flow in the bilateral common carotid arteries also increased. Patients whose symptoms improved postoperatively showed better improvement in the vertebral artery on the VA side. VA flow suppression surgery in patients with high-flow VA increases the flow of the vertebral artery on the VA side and of the bilateral common carotid arteries. High-flow VA can hinder the vertebral and common carotid circulation.

## 1. Introduction

Vascular access (VA) is necessary to achieve sufficient dialysis efficiency in patients undergoing hemodialysis (HD). Arteriovenous fistulae (AVF) and arteriovenous grafts (AVG) are widely recognized as being more favorable than central venous catheters in minimizing the risk of infection [1]. AVF and AVG are categorized as vascular access, causing non-physiological blood flow from arteries to veins. Under normal physiological conditions, arterial blood flows into peripheral resistance vessels, and blood passing through the resistance vessels returns to the heart as venous blood. However, once access is created, arterial blood flows directly into the venous circulation without passing through the resistance vessels. Because the flow resistance of the venous circulation is much lower than that of the peripheral resistance vessels, more arterial blood enters the access than the peripheral circulation. Thus, blood flow in the peripheral artery decreases following the creation of an AVF or AVG. Peripheral circulatory disorders caused by VA are known as access-induced distal ischemia [2]. Coldness, numbness, pain, and finger ulceration due to access-induced distal ischemia have been observed. This phenomenon is a severe complication of VA and is reported to occur at a frequency of 1–8% [3].

Considering access-related systemic circulation changes, not only the peripheral arterial circulation but also all the arterial circulation could decrease due to the difference in flow resistance related to shunt access. The vertebral and common carotid arteries arise from the aorta or the subclavian artery. These arterial circulations may also deteriorate due to the access circulation owing to the difference in flow resistance from the limb artery on the AVF side; the effect may increase as the difference in flow resistance increases. The Japanese guidelines for VA state that a VA flow of more than 1500–2000 mL/min is a risk factor for cardiac failure [1]. In addition to the impact on cardiac function, high-flow VA may significantly hinder other systemic circulations due to extremely low flow resistance. A reverse flow phenomenon in the vertebral artery has been observed in some patients with high-flow access [4]. Subclavian steal syndrome (SSS) is caused by insufficient circulation in the vertebral artery. Although classical SSS occurs because of stenosis or the occlusion of the subclavian artery, SSS without arterial stenosis can be caused by a high-flow VA. It has been reported that VA-induced SSS evokes symptoms such as dizziness more often than SSS with stenosis [4]. Another study reported that VA flow suppression surgery improved SSS due to high-flow AVF [5]; however, the number of such case reports is limited, and the clinical influence of the VA on the vertebral circulation is not fully understood. Furthermore, the effect of VA flow suppression surgery on cervical circulation has rarely been reported.

Therefore, we conducted this study to determine the effects of VA flow suppression surgery on cervical circulation in patients with high-flow VA.

## 2. Materials and Methods

### 2.1. Patients and Study Design

This was a single-center, retrospective, observational study. The study participants were HD patients who underwent VA flow suppression surgery at the Kanno Dialysis and Access Clinic between September 2009 and November 2018. Patients whose cervical arterial flow was measured before and after blood flow suppression surgery were included in this study. VA flow volume (FV) and the FV of the bilateral vertebral and common carotid arteries were measured before and within 1 week after VA flow suppression surgery. Patients with an occluded vertebral or carotid artery were excluded because the pathogenesis of SSS differs with and without obstruction. Patients with insufficient clinical data were excluded. Other patient information, such as age, sex, dialysis vintage, diabetes, and medical cause for VA flow suppression surgery, were obtained from the medical records. Changes in patient symptoms after surgery were determined by checking the medical records.

We also examined the FV of the bilateral common carotid and vertebral arteries in eight healthy volunteers (HVs) and five patients with end-stage kidney disease (ESKD) before creating the VA for comparison.

The study protocol was approved by the ethics committee of Shinshu University (approval number 4631) and was conducted in accordance with the principles of the Declaration of Helsinki, as revised in 2013. Because of the retrospective nature of the study, informed consent was obtained in the form of an opt-out on the web and a poster announcement.

### 2.2. Measurement of Blood FV, Resistance Index (RI), and Cardiac Output (CO)

Blood flow was calculated as the FV based on ultrasound findings. The same ultrasonography equipment (Aplio 500; Toshiba, Tokyo, Japan) was used throughout this study. Pre-surgery measurements were performed 1 day before surgery, whereas post-surgery measurements were performed within 1 week after surgery, both at the time of pre-dialysis. The sonographic parameters were measured by the same technician. The estimated VA flow was calculated as the difference between the VA and non-VA sides of the brachial artery blood flow. The FV of the cervical arteries was measured at the straight part of the bilateral common carotid and vertebral arteries without stenosis. The RI of the brachial artery was also measured. In patients with a high origin of the radial artery, the estimated VA flow was calculated as the difference between the sum of the VA-side radial and ulnar artery flows and the non-VA-side brachial artery flow. These patients were excluded from the RI analysis because they could not be compared to other patients with normal branching patterns.

CO was also measured before and after surgery. CO was calculated using the biplane disk summation method based on a previous report [6]. CO pre-surgery measurements were performed 1 day before surgery, while post-surgery measurements were performed within 1 week after surgery, both at the time of pre-dialysis.

### 2.3. VA Flow Suppression Surgery

VA flow suppression surgery was performed according to the methods described in a previous report [7]. The surgeons selected the appropriate surgical method for each patient. The ligation was performed by ligating and obstructing the target artery using sutures. Banding was performed by narrowing the target artery or runoff vein by wrapping a synthetic graft around the target vessel and suturing the graft. The anastomosis was performed by narrowing the AVF anastomosis site with an exteriorizing anastomosis and suturing. AVG banding was performed by narrowing the synthetic graft via ligation. All VA flow suppression surgical procedures were continued until the VA flow decreased to the target level.

### 2.4. Statistical Analyses

Continuous variables are presented as medians and ranges, and the Mann–Whitney U test was used to compare the two groups. The Wilcoxon signed-rank test was used to compare two paired groups. Categorical variables are presented as percentages. Logistic regression analysis was used for multivariate analysis. Statistical significance was set at *p* < 0.05. The SPSS software (ver. 27; IBM Japan Corp., Tokyo, Japan) was used for statistical analyses. The datasets generated and/or analyzed in this study are available from the corresponding author upon reasonable request.

## 3. Results

**Figure 1:**
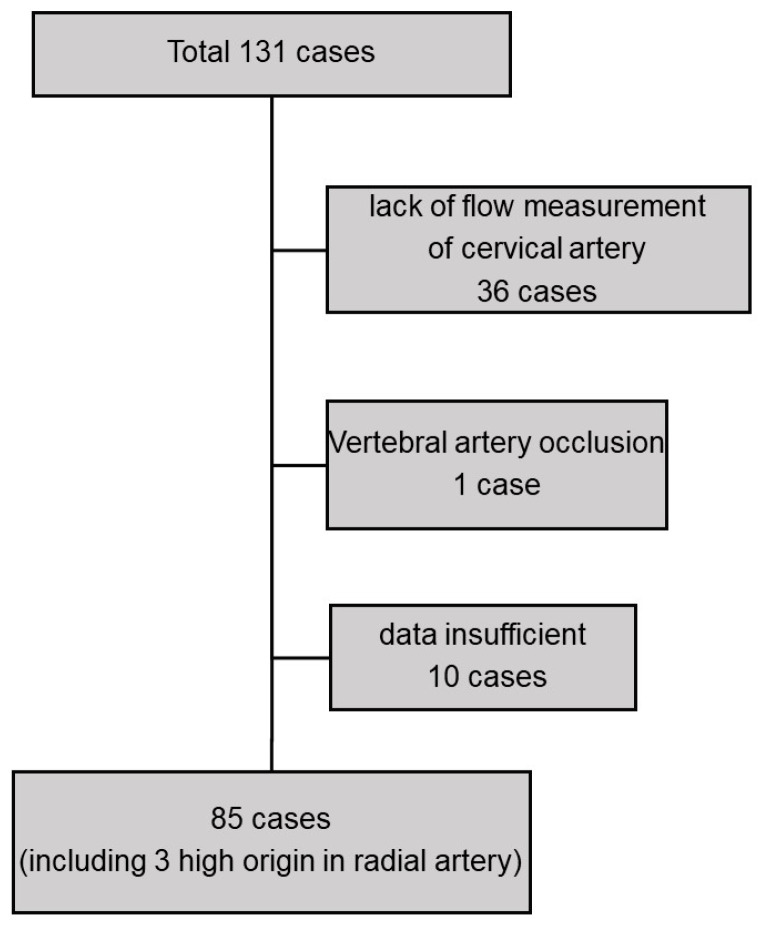
Patient selection.

VA flow suppression surgery was performed in 131 patients during the study period. After applying the exclusion criteria, 85 patients were included in this study (Figure 1). Table 1 presents the patient backgrounds. The median age of the patients was 64 years, and 66% (*n* = 56) were male. Seventy-three percent (*n* = 62) had radiocephalic AVF, and 8% (*n* = 7) had AVG. Eighty-seven percent (*n* = 74) of patients presented with symptoms before the surgery; the most common symptom was exertional breathlessness. The most common reason for VA flow suppression surgery was high-output heart failure. Three patients exhibited high-origin radial artery.

The median VA flow before VA flow suppression surgery was 1548 mL/min (Table 2). The median VA flow decreased significantly to 693 mL/min after surgery. Anastoplasty, proximal artery banding, and distal artery ligation were the most common surgical methods used to suppress the VA flow. The median preoperative RI was 0.44, which increased to 0.54 postoperatively (Table 3). The median CO was 4.5 L/min before the surgery, which significantly decreased to 4.3 L/min postoperatively.

**Figure 2:**
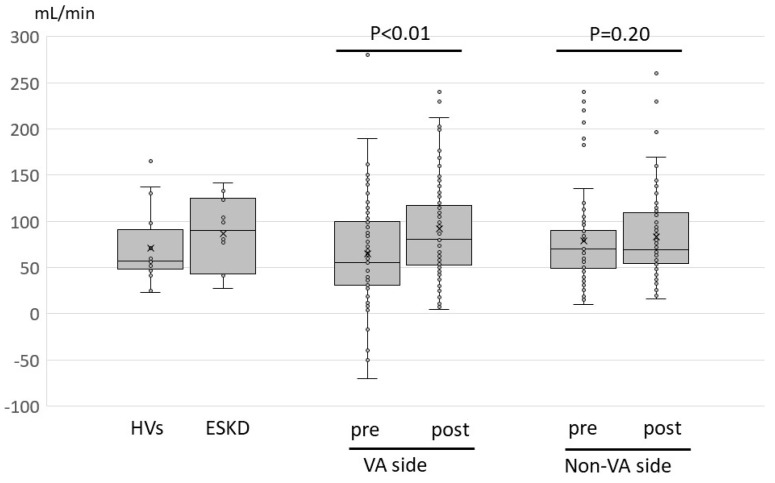
Vertebral artery flow volume of each participant group and changes before and after the surgery. Changes in the vertebral artery flow before and after flow suppression surgery are presented as box-and-whisker plots. Each data is presented as a circle, and the mean value is indicated by x. The *p*-values for comparison between vertebral artery flow of the HVs and VA side or non-VA side of the study participants were 0.49 and 0.37, respectively. The *p*-values for comparison between vertebral artery flow of ESKD patients before VA creation and VA side or non-VA side of the study participants were 0.12 and 0.29, respectively. Abbreviations: HVs, healthy volunteers; ESKD, end-stage kidney disease patients before vascular access creation; VA, vascular access.

**Figure 3:**
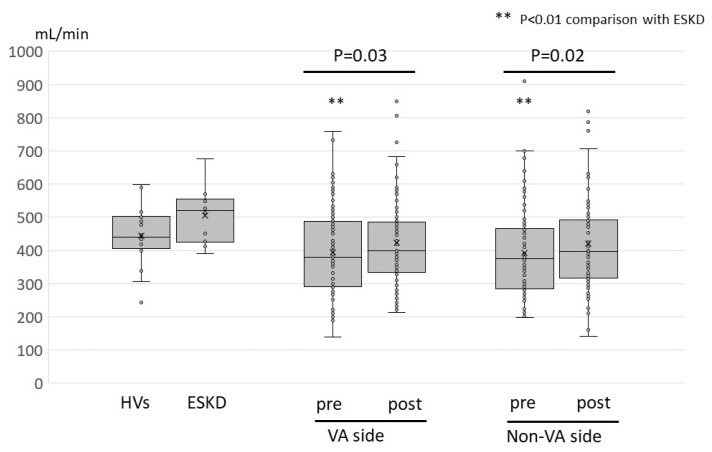
Common carotid artery flow volume of each participant group and changes before and after the surgery. Changes in common carotid artery flow before and after flow suppression surgery are presented as box-and-whisker plots. Each data is presented as a circle, and the mean value is indicated by x. The *p*-values for comparison between the common carotid artery flow of HVs and the VA side or non-VA side of the study participants were 0.06 and 0.05, respectively. The *p*-values for comparison between the common carotid artery flow of ESKD patients before VA creation and the VA side or non-VA side of the study participants were 0.005 and 0.004, respectively. Abbreviations: HVs, healthy volunteers; ESKD, end-stage kidney disease patients before vascular access creation; VA, vascular access.

For flow assessment of the cervical artery, the FV of the vertebral and common carotid arteries are shown in Figure 2 and Figure 3, respectively. The median flow in the vertebral artery in patients with HVs and ESKD was 58 (23–165) mL/min and 90 (27–142) mL/min, respectively (Figure 2). The flows of the VA side vertebral artery in HD patients with high-flow access varied considerably, and four patients (5%) exhibited inverted flow, indicating the subclavian steal phenomenon. The flows of the VA-side vertebral artery were prone to be less than those of the non-VA side; however, the difference was not statistically significant (Table 3). The median flow of the common carotid artery in patients with HVs and ESKD was 440 (244–599) mL/min and 520 (391–677) mL/min, respectively (Figure 3). The median flow in the common carotid artery was identical between the VA and non-VA sides (Table 3). The preoperative flow of the vertebral artery did not differ significantly from that in patients with HVs and ESKD. Meanwhile, the preoperative flows of the common carotid artery were significantly lower than those in patients with ESKD (*p* = 0.004 and *p* = 0.006, respectively) and tended to be lower than those in the HVs (*p* = 0.05, *p* = 0.06, respectively).

After VA flow suppression surgery, the VA-side vertebral artery flow significantly increased from 55 to 81 mL/min. Four patients with inverted flow patterns returned to normal flow patterns after surgery (Figure S1). The vertebral artery flow on the non-VA side did not significantly change postoperatively (Table 3). VA flow suppression surgery also increased the flow volume of the common carotid arteries on both the VA and non-VA side (379 to 398 mL/min and 376 397 mL/min, respectively) (Table 3).

Symptoms improved in 63 of 74 (85%) patients who presented with symptoms before VA flow suppression surgery. Twenty-five (29%) patients presented with neurological symptoms, such as dizziness and/or tinnitus, which could indicate circulatory insufficiency of the cervical artery, and 18 of these patients had improved symptoms postoperatively. When the improved symptoms (n = 18) were compared with the non-improved symptoms (n = 7), the improved symptom group presented a significant increase in flow change in the VA-side vertebral artery (Table 4). The changes in vertebral artery flow in each patient group according to symptoms are presented in Figure S2. The other arterial flows did not differ between the groups (Table 4).

We conducted a multivariate analysis to investigate the factors related to a larger increasing effect on the vertebral artery after surgery. Age, sex, pre-surgical vertebral artery flow, and CO were selected as basic patient characteristics. The VA flow suppression rate was selected as the effect of VA flow suppression surgery. Multivariate analysis revealed that a lower preoperative CO was associated with a larger flow-increasing effect on the vertebral artery (Table 5).

Similarly, we investigated factors related to a larger increase in the common carotid artery after surgery. Multivariate analysis revealed that a lower presurgical FV of the common carotid artery was associated with a larger increase in the flow of the common carotid artery (Table 6).

## 4. Discussion

The current study revealed that VA flow suppression surgery for high-flow access increases the FV of the vertebral and common carotid arteries. Previous studies have reported that high-flow VA can invoke SSS [5,8,9]. Although no obstructions or stenoses existed in the subclavian artery, reversed blood flow of the VA-side vertebral artery was observed in patients with high-flow VA due to the pressure gradient via extremely low VA-side flow resistance. This was a high-flow VA-associated subclavian steal phenomenon.

Previous reports have indicated that stopping VA flow via manual compression or closure of the VA normalized the flow pattern of the vertebral artery in cases of high-flow VA-associated subclavian steal phenomenon [4,8]. In this study, the reversed flow patterns of the vertebral artery were all normalized postoperatively. We observed that patients without reversed flow patterns in the vertebral artery also showed improved FV of the vertebral artery postoperatively. This phenomenon may be due to a decrease in the pressure gradient caused by an increase in the VA flow resistance. Patients in this study did not have a significant decrease in the FV of the VA-side vertebral artery compared with the small number of HVs and ESKD patients. However, blood flow in the vertebral artery varies physiologically among individuals, and the FV of the vertebral artery in patients with HVs and ESKD in this study was widely distributed. The small sample size and wide variation in patients with HVs and ESKD in this study may have prevented the detection of such differences. A previous study measuring the FV of the vertebral artery using sonography in 96 healthy people reported that the mean FV of the vertebral artery was 85 ± 37 mL/min [10], which was larger than the data in this study population. Thus, the blood flow of the vertebral artery in patients with high-flow VA might be lower than that in the healthy population.

Reduced blood flow in the vertebral artery may induce circulatory insufficiency in the vertebrobasilar arterial system. Patients with SSS are prone to dizziness due to vertebrobasilar insufficiency [11]. Symptoms, such as dizziness and tinnitus, were frequently observed in this study population next to heart failure symptoms. When we performed a subgroup analysis of patients with these neurological symptoms before surgery, patients with improved postoperative symptoms exhibited a larger increase in vertebral artery flow than patients with non-improved symptoms. Therefore, reduced blood flow in the vertebral artery could induce neurological symptoms, and VA flow suppression surgery for high-flow VA could improve neurological symptoms via augmentation of vertebral artery flow.

It is known that the cerebral blood flow of HD patients is reduced during HD sessions and is affected by the ultrafiltration volume, filtration rate, and the acid–base balance changes [12]. HD patients with insufficient vertebral artery blood flow due to high-flow VA may exhibit symptoms, such as dizziness due to vertebrobasilar insufficiency, during HD sessions. A previous study reported that patients with symptoms had a high risk of stroke [11]. Thus, HD patients with vertebrobasilar insufficiency symptoms should undergo evaluation of their vertebral artery blood flow and VA flow to prevent future stroke.

Our study also revealed that VA flow suppression surgery could improve vertebral artery blood flow in patients with reduced CO. As the proportion of VA flow to the total circulation was large in patients with high-flow VA, the proportion of other systemic circulations was limited, especially in patients with reduced CO. Thus, patients with reduced CO may have markedly increased systemic circulation, including that of the vertebral artery, after surgery. Patients with a high-flow VA and reduced CO may benefit more from surgery.

This study also revealed that blood flow in the common carotid artery improved postoperatively. To the best of our knowledge, this is the first report describing the impact of VA flow suppression surgery on the common carotid artery. When the flow resistance of the VA is significantly lower than that of the systemic circulation, various parts of the circulatory system with flow resistance higher than that of the VA may be affected. The common carotid artery is thicker than the vertebral artery, its flow resistance is lower than that of the vertebral artery, and its blood flow is higher. Thus, because the vertebral arteries inherently have less blood flow than the common carotid arteries, attention tends to focus on the effects on the vertebral arteries, which are more prone to malperfusion symptoms when blood flow is reduced. However, the current results showed that the FV of the common carotid artery was also affected by high-flow VA, which was less than that in patients with ESKD, and was improved by VA flow suppression surgery. Further research is required to verify whether the impact of high-flow VA on the flow of the common carotid artery causes any clinically relevant problems.

This study has several limitations. First, this was a single-center retrospective study, and we could not examine parameters that were not recorded. Therefore, we cannot exclude the effects of uncoordinated confounders. Second, all patients included in this study underwent blood flow suppression surgery for the treatment of high-flow VA; therefore, a selection bias may have been present when deciding on VA flow suppression surgery. Additional studies are required to demonstrate the impact of normal-flow VA on cervical circulation. Third, because the flow volumes of the VA, vertebral artery, and common carotid artery before and after VA flow suppression surgery were measured at one point, it is unclear how long this effect lasts for cervical circulation. A prospective observational study with a longer observation period is required to confirm this finding. Fourth, this was an observational study with no control group, and changes in the symptoms of the patients were examined through interviews with medical staff related to the VA flow suppression surgery. Therefore, the results of this study relating to symptom changes after surgery may have contained information bias.

## 5. Conclusions

In conclusion, VA flow suppression surgery in patients with high-flow VA increased blood flow to the VA-side vertebral artery and bilateral common carotid arteries along with improving various symptoms caused by high flow volume. High-flow VA can decrease the vertebral and common carotid circulation. Medical staff involved in HD should measure VA flow not only in patients with VA troubles but also in patients who present with neurological symptoms such as dizziness or reduced CO. When the measured VA flow is large, medical staff should consider performing VA flow suppression surgery.

## Figures and Tables

**Table 1:** Patient characteristics.

Characteristics	
**No. of patients**	85
**Age (years), *n* (range)**	64 (28–87)
**Male, *n* (%)**	56 (66%)
**Dialysis vintage (month), m (range)**	88 (3–1341)
**Diabetes mellitus, *n* (%)**	11 (13%)
**Left side access, *n* (%)**	72 (85%)
**Access type, *n* (%)**	
Radiocephalic AVF	62 (73%)
Ulnar-basilic AVF	1 (1%)
Brachiocephalic AVF	15 (18%)
AVG	7 (8%)
**Symptoms, *n* (%)**	
Exertional breathlessness	51 (60%)
Palpitation	5 (6%)
Distal coldness	10 (12%)
Venous hypertension	12 (14%)
Dizziness	14 (16%)
Tinnitus	9 (11%)
Other neurological symptoms	5 (6%)
Other	4 (5%)
No symptoms	11 (13%)
**Reasons for needing blood suppression, *n* (%)**	
High-output heart failure	50 (59%)
Low cardiac function	10 (12%)
Venous hypertension	12 (14%)
Access vessel aneurysm	12 (14%)
Distal steal syndrome	8 (9%)
Subclavian steal syndrome	4 (5%)
Pulmonary hypertension	1 (1%)

Continuous variables are expressed as medians and ranges. Categorical variables were expressed as numbers and percentages. Abbreviation: AVF, arteriovenous fistula; AVG arteriovenous graft.

**Table 2:** Surgical methods for blood flow suppression and changes in the VA flow.

	*n* (%)	VA Flow (pre) (mL/min)	VA Flow (post) (mL/min)	*p*	Reduction Rate (%)
All cases	85	1548 (649–2453)	693 (321–1265)	<0.01	53 (18–83)
Surgical methods					
Proximal artery ligation	13 (15%)	1619 (891–2198)	514 (321–995)	<0.01	57 (29–83)
Proximal artery banding	10 (12%)	1396 (1114–2006)	621 (413–1248)	<0.01	57 (18–65)
Proximal artery banding and distal artery ligation	20 (24%)	1561 (1048–2453)	703 (353–1063)	<0.01	56 (29–78)
Anastoplasty	23 (27%)	1584 (649–2447)	723 (357–1248)	<0.01	48 (25–83)
Run-off vein banding	4 (5%)	1354 (904–2143)	666 (353–1265)	<0.01	54 (26–63)
AVG banding	6 (7%)	1252 (1036–1610)	580 (380–801)	<0.01	56 (34–72)
Other	9 (11%)	1633 (1192–2065)	859 (442–1208)	0.03	43 (20–72)

Continuous variables are expressed as medians and ranges. Categorical variables were expressed as numbers and percentages. Preoperative and postoperative differences were compared using the Wilcoxon signed-rank test. Other: Revision using distal inflow (RUDI), 3; distal radial artery ligation, 3; runoff vein ligation, 3. Abbreviations: VA, vascular access; AVG, arteriovenous graft

**Table 3:** Changes in the VA-related parameters and flow volume of cervical arteries.

Parameters	Pre-Surgery	Post-Surgery	*p*
Resistance index (RI)	0.44 (0.22~0.64)	0.54 (0.39~0.83)	<0.01
Cardiac output (CO) (L/min)	4.5 (2.4~9.9)	4.3 (2.3~8.6)	<0.01
Vertebral artery			
VA side flow (mL/min)	55 (−70~280)	81 (5~370)	<0.01
Non-VA side flow (mL/min)	70 (10~240)	69 (16~260)	0.20
Common carotid artery			
VA side flow (mL/min)	379 (140~759)	398 (212~850)	0.03
Non-VA side flow (mL/min)	376 (198~910)	397 (141~820)	0.02

Continuous variables are expressed as medians and ranges. Preoperative and postoperative differences were compared using the Wilcoxon signed-rank test.

**Table 4:** Comparison between the neurological symptom improved group and non-improved group.

	Symptoms Improved	Non-Improved	*p*
*n* (%)	17 (68%)	8 (32%)	
Flow reduction rate, *n* (%)	58.5 (34~78)	61.0 (25~77)	1.00
Vertebral artery			
VA side flow change (mL/min)	48 (−53~160)	2 (−81~90)	0.02
Non-VA side flow change (mL/min)	5 (−48~90)	6 (−30~46)	0.84
Common carotid artery			
VA side flow change (mL/min)	20 (−88~210)	−6 (−100~360)	0.75
Non-VA side flow change (mL/min)	11 (−83~250)	119 (−203~261)	0.37

Continuous variables are expressed as medians and ranges. Categorical variables are expressed as numbers and percentages. Differences between groups were compared using the Mann–Whitney U test. Abbreviations: VA; vascular access.

**Table 5:** Univariate and multivariate analyses for the flow-increasing effect in the vertebral artery after flow suppression surgery.

Parameters	Univariate			Multivariate		
	Odds Ratio	CI	*p*	Odds Ratio	CI	*p*
Age	1.01	0.97–1.04	0.75	1.00	0.96–1.05	0.86
Male	1.15	0.47–2.82	0.76	1.46	0.54–3.89	0.46
Flow reduction rate	1.02	0.99–1.05	0.30	1.02	0.99–1.01	0.99
Pre-Vertebral A flow	1.00	0.99–1.01	0.56	1.00	0.99–1.01	0.49
Pre-CO	0.74	0.54–1.03	0.07	0.70	0.49–0.99	0.04

Factors related to a larger flow-increasing effect in the vertebral artery were analyzed using logistic regression analysis. Abbreviations: Pre-CO, presurgical cardiac output; CI, confidence interval.

**Table 6:** Univariate and multivariate analyses for the flow-increasing effect in the common carotid artery after flow suppression surgery.

Parameters	Univariate			Multivariate		
	Odds Ratio	CI	*p*	Odds Ratio	CI	*p*
Age	1.00	0.97–1.03	0.98	0.99	0.95–1.03	0.56
Male	0.61	0.25–1.51	0.29	0.86	0.31–2.37	0.77
Flow reduction rate	1.01	0.98–1.04	0.49	1.02	0.99–1.05	0.24
Pre-CCA flow	0.99	0.99–1.00	<0.01	0.99	0.98–0.99	0.01
Pre-CO	0.80	0.58–1.09	0.15	0.90	0.63–1.29	0.57

Factors related to a larger flow-increasing effect in the common carotid artery were analyzed using logistic regression. Pre-CCA, pre-surgical common carotid artery; pre-CO, pre-surgical cardiac output; CI, confidence interval.

## Data Availability

Data are contained within the article and supplementary materials.

## Supplementary Materials

The following supporting information can be downloaded at: https://www.mdpi.com/article/10.3390/jcm13030641/s1, Figure S1: Flow pattern change in the vertebral artery before and after VA flow suppression surgery; Figure S2: (A) Changes in VA side vertebral artery flow volume of each patient group according to the symptoms; (B) Changes in non-VA side vertebral artery flow volume of each patient group according to the symptoms.
