# Supplementary material for: Impact of Vascular Access Flow Suppression Surgery on Cervical Artery Circulation: A Retrospective Observational Study
Source: J Clin Med. 2024 Jan 23;13(3):641. doi: 10.3390/jcm13030641 (PMC10856206; doi:10.3390/jcm13030641)
Supplement: Supplementary file 1 [file jcm-13-00641-s001.zip › jcm-2819126-supplementary.pdf]

Journal of
Clinical Medicine

MDPI

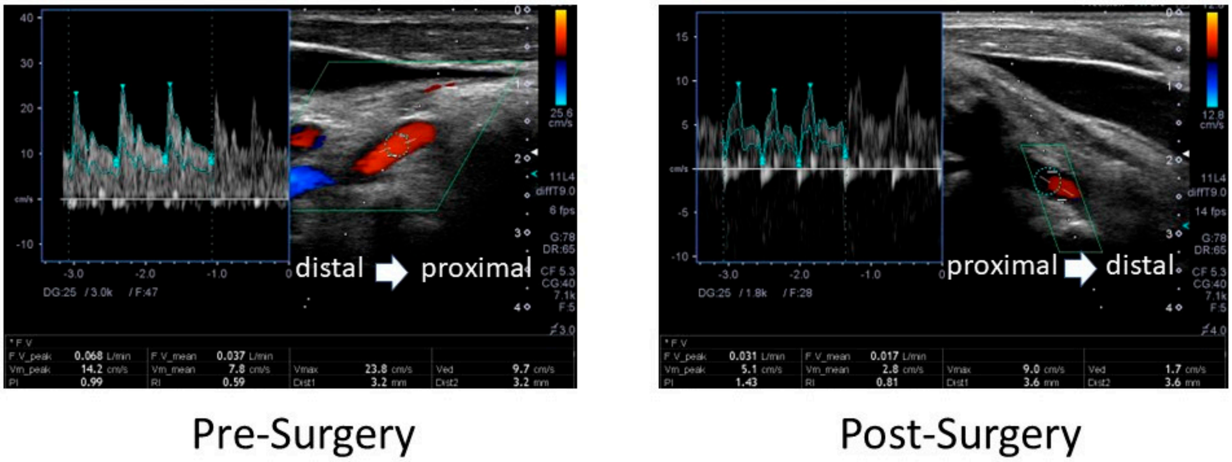

Figure S1. Flow pattern change in the vertebral artery before and after VA flow suppression surgery. The flow direction of the vertebral artery was inverted before vascular access flow-suppression surgery y(left). The flow direction improves to a normal pattern after surgery (right). VA, vascular access.

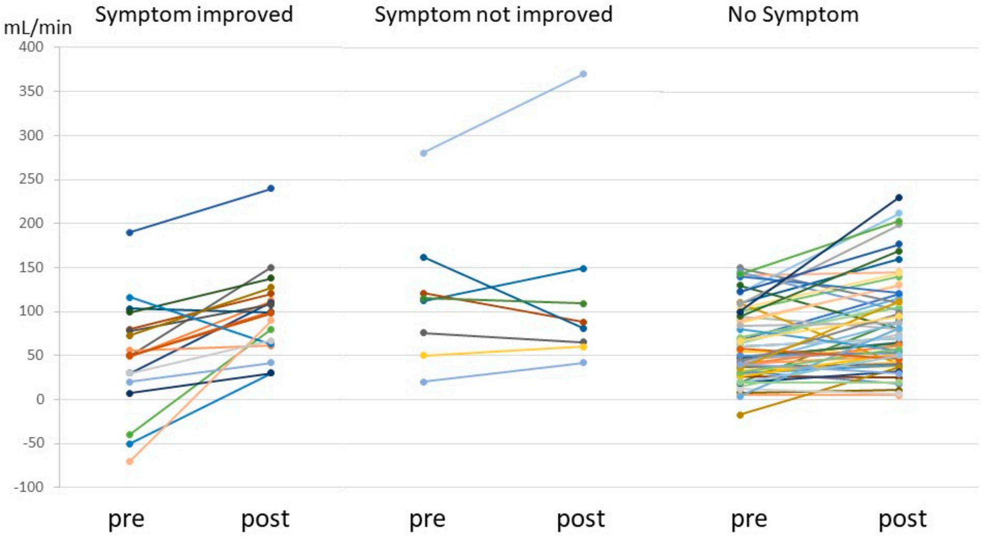

Figure S2A. Changes in VA side vertebral artery flow volume of each patient group according to the symptoms.

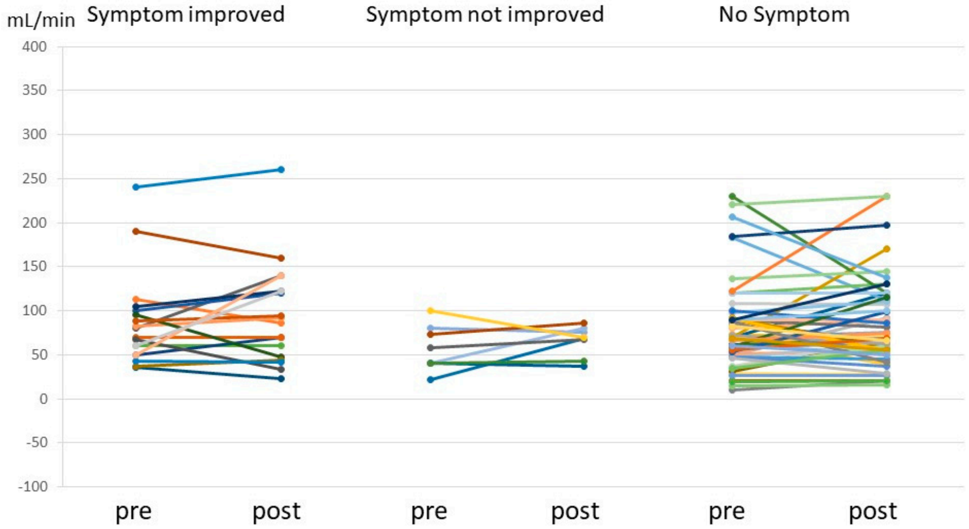

Figure S2B. Changes in non-VA side vertebral artery flow volume of each patient group according to the symptoms. Patients who presented with neurological symptoms and whose symptoms improved postoperatively had significantly increased blood flow in the vertebral artery on the VA side compared with the group of patients who did not; however, this phenomenon was not observed on the non-VA side. VA, vascular access.
